# The Big Data Era in Cardiology and Cardiovascular Medicine: Advanced Analytics for Truly Personalized Care

**DOI:** 10.3390/diagnostics15131705

**Published:** 2025-07-03

**Authors:** Pasquale Ambrosino, Fabio Manzo, Claudio Candia, Giorgio Alfredo Spedicato, Guido Grassi, Mauro Maniscalco

**Affiliations:** 1 Istituti Clinici Scientifici Maugeri IRCCS, Scientific Directorate of Telese Terme Institute, 82037 Telese Terme, Italy; 2 Fleming Clinical Laboratory, 81020 Casapulla, Italy; fabioce26@gmail.com; 3 Department of Biomedicine, Neurosciences and Advanced Diagnostics, University of Palermo, 90100 Palermo, Italy; c.candia@outlook.it; 4 Department of Statistics and Quantitative Methods, Milano-Bicocca University, 20126 Milan, Italy; spedicato_giorgio@yahoo.it; 5 Department of Medicine and Surgery, Milano-Bicocca University, 20126 Milan, Italy; 6 Istituti Clinici Scientifici Maugeri IRCCS, Pulmonary Rehabilitation Unit of Telese Terme Institute, 82037 Telese Terme, Italy; mauro.maniscalco@icsmaugeri.it; 7 Department of Clinical Medicine and Surgery, Federico II University, 80131 Naples, Italy

In recent years, a growing body of alarming epidemiological evidence has suggested that cardiovascular diseases (CVDs) are the leading cause of disability and premature mortality worldwide. According to estimates from the Global Burden of Disease (GBD) study, CVDs were responsible for approximately 19 million deaths globally in 2021 and accounted for 438 million disability-adjusted life years (DALYs) lost [1]. This causes a profound impact on healthcare systems and national economies worldwide, posing an increasingly urgent challenge for both the scientific community and public health authorities [2]. The situation is further exacerbated by discouraging future projections, which anticipate a continued rise in prevalence, mortality, and DALYs between 2025 and 2050, a trend largely driven by the aging global population [3]. These alarming forecasts highlight the pressing need for two complementary approaches, the first involving the implementation of broad population-based prevention strategies, and the second focusing on the promotion of increasingly personalized pharmacological and rehabilitation interventions tailored to the specific needs of individual patients [4].

Although the ultimate goal of personalized medicine is to deliver the right treatment to the right patient [5], its implementation in cardiovascular care is still limited by the fact that most clinical guidelines are derived from evidence based on the “average patient” model [6]. Randomized controlled trials (RCTs), for instance, typically report aggregate outcomes across entire study populations and only subsequently attempt to assess the heterogeneity of treatment effects using traditional subgroup analyses, with forest plots stratifying results by single clinical or demographic variables. While this approach provides a valuable overview, it fails to fully account for the true clinical complexity of individual patients and the interplay of multiple variables coexisting across different patient clusters [4]. In this context, advanced statistical tools and top-tier evidence, including meta-analyses of RCTs, can offer more robust and consistent insights. However, while methodologically sound and effective at the population level, this approach remains fundamentally distant from the core principles of precision medicine [5]. Truly personalized care comes from personalized diagnostics, which requires a deep understanding of the biological and molecular mechanisms underlying disease processes and their phenotypic expression in different individuals [6]. Precision medicine, therefore, is not merely about stratifying existing data, but about integrating molecular (genomic, transcriptomic, proteomic, metabolomic), phenotypic, and clinical data to generate patient-specific approaches, thus improving both prevention and treatment strategies while enhancing health outcomes [7]. In recent years, the field of omics has made significant progress, laying the foundation for future generations to benefit from truly personalized medicine. However, this approach is still often inaccessible due to its high costs, advanced technical requirements, and limited feasibility, especially in resource-limited contexts [8].

Concurrently, healthcare systems are being reshaped by several changes, many of which have been facilitated by the COVID-19 pandemic. Specifically, this global crisis induced governments around the world to step up their digital transformation efforts, promoting telemedicine and patient electronic monitoring, processes that would have otherwise taken much longer to implement [9]. Therefore, with massive volumes of health information collected from electronic health records (EHRs), the healthcare industry is now uniquely positioned to benefit from data-driven approaches to guiding innovation and improving care at both the individual and population levels [10]. Data from EHRs are a relatively recent development and should be integrated with complementary data sources, including administrative databases, imaging data, patient-reported outcomes, and environmental or socioeconomic data [11]. An example of such integration is the TriNetX platform, which was launched in 2014 and is now powered by real-world data from nearly 300 million patients across more than 30 countries, sourced from EHRs, claims, and other databases from hundreds of healthcare organizations [12]. This network represents a unique opportunity to enhance the optimization of RCTs and facilitate large-scale observational studies, particularly in the cardiovascular field [12]. Numerous articles based on thousands of patient records have been recently published utilizing the TriNetX network, with a particular focus on the clinical efficacy of novel cardiovascular therapies such as glucagon-like peptide-1 (GLP-1) receptor agonists [13,14]. These investigations aim to employ large-scale real-world data to rapidly address clinical questions on novel pharmacological and rehabilitation approaches that traditionally would have required epidemiological evidence collected over many years. Additionally, the platform has contributed to recent findings regarding a reduced incidence of CVD following herpes zoster vaccination [15], the underdiagnosis of cardiac sarcoidosis in young patients with unexplained heart block [16], and the role of routine cardiac biomarkers in predicting major adverse cardiac events in patients with glomerulonephritis [17].

In this evolving landscape, a growing number of clinical registries, promoted by scientific societies since the 1980s, further contributes to the expanding volume of large-scale health information now available in the digital format [11]. Some of the most representative CVD registries currently in use have been developed through the initiatives of the American College of Cardiology (ACC), the Society of Thoracic Surgeons (STS), the American Heart Association (AHA), and the American Stroke Association (ASA) [18]. Among these, the Transcatheter Valve Therapy (TVT) registry plays an important role in collecting and analyzing data on patients undergoing transcatheter valvular procedures [19]. With millions of patient records and hundreds of high-profile studies, the largest-scale initiative remains the National Cardiovascular Data Registry (NCDR) with its sub-registries, created by the ACC in 1987 to describe the clinical characteristics and outcomes of patients undergoing cardiac catheterization and coronary interventions [20]. Similarly, the Get With The Guidelines (GWTG) Database is another hospital-based initiative led by the AHA and the ASA, collecting data from thousands of hospitals to improve diagnostics and quality of care in CVDs, such as stroke, atrial fibrillation, and heart failure [21].

Alongside this vast amount of data collected for professional and scientific purposes, the massive volume of information collected from outside healthcare systems through smartphones and wearable technologies is increasingly facilitating the continuous monitoring of lifestyle and cardiovascular parameters, including heart rate, blood pressure, oxygen saturation, sleep quality or even electrocardiographic (ECG) data [22]. Therefore, a number of large-scale studies have been initiated, including the Apple Heart Study, which investigated the ability of optical sensors embedded in a commercial smartwatch to detect atrial fibrillation in real-world settings [23]. This shift toward real-time, longitudinal health monitoring represents a potentially revolutionary step forward, especially in cardiovascular care, due to the nature of the parameters most easily captured by wearable devices [24].

Overall, while omics technologies are not yet widely available or fully integrated into clinical practice [8], “big data” derived from EHRs and other complementary sources now represent a solid base for generating predictive models and identifying patterns [25], supported by the large amount of observations and increasingly sophisticated analytical methods. Big data, in fact, usually describes extremely large and complex datasets that exceed the capacity of conventional tools and processing infrastructures to effectively manage and analyze information. Artificial intelligence (AI), including machine learning and deep learning techniques, as well as data science approaches have recently shown potential in transforming risk assessment, early diagnosis, and the personalization of therapy in cardiovascular medicine [26]. In fact, machine learning models can capture complex nonlinear relationships and interactions among variables, perform effective feature selection, and offer interpretability through specific tools (e.g., Shapley values), which reduce the perception of opacity often associated with these approaches [27]. Additionally, given the multifactorial nature of CVDs, multimodal deep learning models even enable the integration of different data sources in different formats, such as images, ECG sequences, and tabular clinical data, thus facilitating a unified analysis of heterogenous cardiac information to improve diagnostic and prognostic assessments [27].

Traditionally, imaging and ECG signal interpretation have represented the earliest and most successful areas of integration between AI and cardiovascular medicine, supporting early detection while minimizing scan duration and exposure to ionizing radiation [28]. Since the first algorithm received regulatory clearance in 1995, the Food and Drug Administration has approved more than 60 clinical AI applications specifically for cardiovascular medicine, out of a total of over 600 approvals, with the majority granted in the past five years [26]. Thus, as coronary artery calcium scoring becomes a key tool in cardiovascular risk stratification, AI algorithms are now able to automatically quantify calcium scores not only from low-dose chest computed tomography (CT) scans, but also from nuclear imaging techniques such as positron emission tomography (PET)/CT [28]. AI-assisted applications have also been successfully implemented in the volumetric and structural analysis of cardiac chambers using cardiac magnetic resonance (CMR) [29], as well as in guiding procedures for the ablation of infarct-related ventricular tachycardia [30]. Similarly, AI has been used in electrocardiography to diagnose atrial fibrillation or predict imminent ventricular arrhythmias [31], in coronary angiography to identify stenosis from angiograms [29], and in echocardiography to automatically evaluate valvular structures and flow gradients, calculate ejection fraction, measure longitudinal strain, and detect abnormalities in cardiac wall motion [28]. The Digital Aortic Stenosis Severity Index (DASSi) is an example of an AI tool that can not only recognize severe aortic stenosis from a single echocardiographic image but also stratify individuals based on their future risk of disease onset or rapid progression [32]. Models based on more computationally intensive methods, such as boosting algorithms (e.g., gradient boosting machines, LightGBM, XGBoost) and deep learning architectures, have been applied to EHRs in various clinical studies to predict the risk of incident hypertension, heart failure readmissions, and ischemic stroke [28,31]. Moreover, the opportunities introduced by virtual reality and wearable technologies are stimulating increasing interest in the use of AI to monitor and support physical exercise within both home-based and in-hospital rehabilitation programs [33,34,35].

Overall, as we gradually approach the era of clinical omics, we already possess an enormous volume of heterogeneous electronic information from a wide range of sources. The central challenge in this evolving landscape is achieving effective data integration and interoperability across systems and platforms. Although important concerns regarding privacy and equity persist, this is precisely where AI and advanced computational approaches are beginning to make a significant difference, helping to extract and unify complex datasets and drive the transition toward truly individualized, data-informed cardiovascular diagnostics and care.

## Data Availability

No datasets were generated or analyzed during the current study.
